# Brugada-Phenocopy Induced by Propafenone Overdose and Successful Treatment: A Case Report

**DOI:** 10.4274/balkanmedj.2016.1185

**Published:** 2017-09-29

**Authors:** Mehmet Emre Arı, Filiz Ekici

**Affiliations:** 1 Department of Pediatric Cardiology, Ankara Children’s Hematology and Oncology Training and Research Hospital, Ankara, Turkey

**Keywords:** Brugada-like electrocardiography pattern, propafenone, children, Brugada phenocopy

## Abstract

**Background:**

Brugada syndrome is an inherited arrhythmogenic disease that may cause sudden cardiac death due to ventricular fibrillation in young adults. Brugada syndrome caused by propafenone intoxication has been noted rarely in the literature. We report a rare case, Brugada phenocopy due to propafenone intoxication and its treatment.

**Case Report:**

A 15-year-old girl having a seizure was brought to the emergency room. She took 1.5 g propafenone (Rythmol, Abbott, Chicago, IL, USA) for suicidal intention. She had metabolic acidosis. Long QRS interval and ST elevation in leads V_1_ through V_3_ were seen on electrocardiography. After bicarbonate infusion for 4 hours, haemodynamic and neurologic findings were recovered, and all electrocardiography abnormalities disappeared. The Brugada-like electrocardiography pattern was not recognized in her surface and 24-hour Holter electrocardiography at follow-up. Ajmaline challenge test was negative 2 weeks later.

**Conclusion:**

Absence of symptoms and documented ventricular tachycardia, negative challenge test, and a negative family history demonstrated that the Brugada phenocopy was a transient finding in this case and related to propafenone intoxication.

Brugada syndrome is an inherited arrhythmogenic disease that may cause sudden cardiac death due to ventricular fibrillation in young adults. Brugada syndrome is usually identified by a characteristic type 1 electrocardiography (ECG) pattern that consists of ST elevation of a coved type in the precordial leads V_1_ to V_3_. In addition, the term Brugada phenocopy is used to describe conditions that induce Brugada-like ECG manifestations in patients without true Brugada syndrome. Some drugs and conditions can cause a Brugada type 1 ECG pattern which is not true congenital Brugada syndrome (1,2).

Propafenone (Rythmol, Abbott, Chicago, IL, USA) is a group 1C antiarrhythmic drug that has sodium channel-blocking effects. Excessive intake may cause severe arrhythmias such as bradycardia, sinoatrial block, atrioventricular block, intraventricular block, junctional and/or ventricular tachycardia, or chaotic ventricular rhythm (3,4,5,6,7). Intoxication with class I antiarrhythmic drugs is associated with a higher mortality rate (22.5%) than other drugs (1%) (3).

We present a case report of successful treatment of Brugada phenocopy due to propafenone intoxication.

## CASE PRESENTATION

**FIG. 1.**
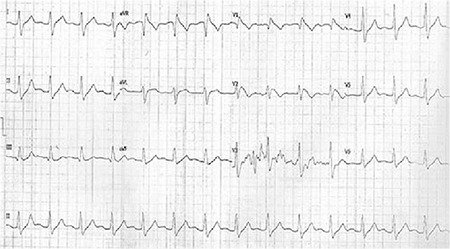
Brugada phenocopy appeared in leads V_1_ through V_3_.

**FIG. 2.**
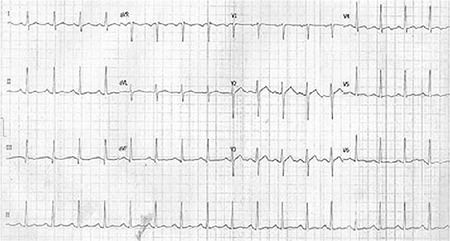
All electrocardiography abnormalities returned to normal 4 hours later.

A 15-year-old girl was admitted to the emergency room with complaints of vomiting and loss of consciousness lasting 3 hours. Upon admission, her blood pressure was 70/45 mmHg, her heart rate was 60/minute, and the body temperature was 36.8 °C. The initial ECG revealed sinus rhythm, absent of the P-wave, and an extremely prolonged QRS interval (280 ms). It was reported that she had attempted suicide by taking five pills of propafenone hydrochloride (total dose 1.5 g, 30 mg per kilogram of body weight) 3 hours before admission. Generalized tonic-clonic convulsion was observed in the patient and treated with midazolam infusion. Arterial blood gas analysis revealed mild metabolic acidosis. The other laboratory tests showed that blood glucose level, electrolytes, hepatic and kidney function tests, and troponin 1 levels were normal. Bedside echocardiographic examination was normal as well. Intravenous sodium bicarbonate, saline, and positive inotropic agent infusions were started immediately. On a following ECG, Brugada phenocopy appeared in leads V_1_ through V_3_ (Figure 1). Haemodynamic and neurologic findings were recovered 4 hours after treatment. When the acidosis was treated, the ECG abnormalities returned to normal (Figure 2). After the treatment was completed, 24 hour rhythm Holter ECG recording was normal. The patient was discharged from hospital without any sequel 2 days after admission. We observed that ajmaline challenge test was negative 2 weeks after the incident. She had no medication and arrhythmic syncope in the history. Her family members had neither pathological ECG findings nor history for sudden cardiac death. We have received confirmation from the family to be published.

## DISCUSSION

The characteristic ECG irregularities of Brugada syndrome may be seen intermittently. It can be unmasked by sodium channel blockers and febrile states. In addition, some drugs and conditions can trigger a Brugada type 1 ECG pattern without true congenital Brugada syndrome. The terminology of this situation in the literature is diverse and variable such as acquired forms of Brugada syndrome, Brugada-like ECG patterns, Brugada-like ECG findings, Brugada-like ECG ST segment abnormalities, and Brugada syndrome mimicry (1,2,8). The lack of consensus on the terminology is confusing. As such, we need to use a common term for this Brugada syndrome type. Baranchuk et al. (1) suggested that Brugada phenocopy is the best descriptor for Brugada-like ECG patterns in patients without true Brugada syndrome. A type 1 ECG pattern of Brugada syndrome was observed in our patient, who did not meet any clinical or ECG criteria. Thus, it was accepted as propafenone-induced Brugada phenocopy. Similarly, Hasdemir et al. (6) presented a 16-year-old girl who took 2.4 g propafenone and had extreme QRS complex widening and prolonged PR interval on ECG. Brugada phenocopy was seen on ECG after saline infusion therapy, and she was discharged from hospital without any sequel.

Metabolic conditions (hypokalaemia, hyperkalaemia, hyponatraemia, and hypercalcaemia), medicines with a sodium channel-blocking effect such as class 1 antiarrhythmic drugs, anaesthetics (especially propofol), tricyclic antidepressants, severe fever, or cocaine can cause Brugada phenocopy (1,3,4,5,6,7). Junttila et al. (7) evaluated 47 patients with a typical Brugada-type ECG due to medicines or severe fever in the emergency room. They detected malignant arrhythmias and sudden cardiac death in 51% and 37% of cases respectively.

There is no specific treatment for propafenone intoxication. Haemodialysis or haemoperfusion can be used, but are frequently unsuccessful. Atropine, inotropic drugs, or pacemaker therapy may be required. As in our case, intravenous sodium bicarbonate infusion may support rapid and full recovery of dysrhythmia related to propafenone intoxication (3,4,5,6,7). If propafenone-induced cardiotoxicity is unresponsive to the current treatment, intravenous lipid emulsion therapy or glucose-insulin infusion may be considered.

In conclusion, Brugada phenocopy is the best term to define the Brugada type 1 ECG pattern without congenital Brugada syndrome. We presented a very rare case in children, propafenone intoxication which causes Brugada phenocopy.
